# Virulence and resistance patterns of *Vibrio cholerae* non-O1/non-O139 acquired in Germany and other European countries

**DOI:** 10.3389/fmicb.2023.1282135

**Published:** 2023-11-22

**Authors:** Katarzyna Schmidt, Holger C. Scholz, Sandra Appelt, Jana Michel, Daniela Jacob, Susann Dupke

**Affiliations:** ^1^Mycotic and Parasitic Agents and Mycobacteria, Robert Koch Institute, Berlin, Germany; ^2^ECDC Fellowship Programme, Public Health Microbiology Path (EUPHEM), European Centre for Disease Prevention and Control (ECDC), Stockholm, Sweden; ^3^Centre for Biological Threats and Special Pathogens, Highly Pathogenic Microorganisms (ZBS 2), Robert Koch Institute, Berlin, Germany

**Keywords:** *Vibrio cholerae* non-O1/non-O139, clinical and environmental isolate, whole genome sequencing, antimicrobial resistance genes, virulence genes, core genome multilocus sequence typing (cgMLST)

## Abstract

Global warming has caused an increase in the emergence of *Vibrio* species in marine and estuarine environments as well as fresh water bodies. Over the past decades, antimicrobial resistance (AMR) has evolved among *Vibrio* species toward various antibiotics commonly used for the treatment of *Vibrio* infections. In this study, we assessed virulence and resistance patterns of *Vibrio cholerae* non-O1/non-O139 strains derived from Germany and other European countries. A total of 63 clinical and 24 environmental *Vibrio cholerae* non-O1/non-O139 strains, collected between 2011 and 2021, were analyzed. *In silico* antibiotic resistances were compared with resistance phenotypes according to EUCAST breakpoints. Additionally, genetic relatedness between isolates was assessed by two cgMLST schemes (SeqSphere +, pubMLST). Both cgMLST schemes yielded similar results, indicating high genetic diversity among *V. cholerae* non-O1/non-O139 isolates. Some isolates were found to be genetically closely related (allelic distance < 20), which suggests an epidemiological link. Thirty-seven virulence genes (VGs) were identified among 87 *V. cholerae* non-O1/non-O139 isolates, which resulted in 38 virulence profiles (VPs). VPs were similar between clinical and environmental isolates, with the exception of one clinical isolate that displayed a higher abundance of VGs. Also, a cluster of 11 environmental isolates was identified to have the lowest number of VGs. Among all strains, the predominant virulence factors were quorum sensing protein (*luxS*), repeats-in-toxins (*rtxC/rtxD*), hemolysin (*hlyA*) and different type VI secretion systems (T6SS) genes. The genotypic profiles revealed antibiotic resistance genes (ARGs) associated with resistance to beta-lactams, quinolones, macrolides, tetracycline, antifolate, aminoglycosides, fosfomycin, phenicols and sulfonamide. Carbapenemase gene VCC-1 was detected in 10 meropenem-resistant *V. cholerae* non-O1/non-O139 isolates derived from surface water in Germany. The proportion of resistance among *V. cholerae* non-O1/non-O139 species isolates against first line treatment (3rd generation cephalosporin, tetracycline and fluoroquinolone) was low. Empirical treatment would likely have been effective for all of the clinical *V. cholerae* non-O1/non-O139 isolates examined. Nevertheless, carbapenem-resistant isolates have been present in fresh water in Germany and might represent a reservoir for ARGs. Monitoring antimicrobial resistance is crucial for public health authorities to minimize the risks for the human population.

## 1 Introduction

*Vibrio* species are aquatic gram-negative bacteria that are found worldwide in fresh and coastal marine waters with moderate salinity. *Vibrio cholerae* serogroup O1 and O139 strains are well known to be the causative agent of endemic and epidemic cholera. Cholera is occasionally diagnosed in Europe as a travel-associated infection, but not endemic. Pathogenic *Vibrio cholerae* O1/O139 strains encode two major virulence factors: enterotoxin (cholera toxin CT; product of the *ctxA*, *ctxB* genes) and the toxin coregulated pilus (colonization factor TCP: *tcpA*), both of which enable bacterial cells to adhere and colonize the small intestine. The expression of both virulence factors causes severe diarrhea with the risk of dehydration. The major virulence genes are present in clusters and are encoded on mobile genetic elements integrated into the chromosomes of the toxigenic strains. The CTX genes are coded by part of the genome of a filamentous bacteriophage (CTXΦ), while TCP genes are located within a pathogenicity island (VPI).

There are several species of non-cholera *Vibrio*, often called “non-toxigenic” due to the lack of CT and TCP virulence genes. These include *V. cholerae* strains not previously mentioned, and other *Vibrio* species. However, other virulence factors, previously found in the toxigenic strains, like type III and type VI secretion systems (T3SS, T6SS), mannose-sensitive hemagglutinin pilus (*mshA*), thermostable hemolysin (*hlyA*), outer membrane proteins (*ompU*), cholix toxin (*chxA*) and repeats-in-toxin (RTX) clusters (Dziejman et al., 2005; Chatterjee et al., 2009; Arteaga et al., 2020) are known to contribute to their pathogenicity. The occurrence of those virulence genes varies between the strains. The clinical manifestation of non-toxigenic *Vibrio* species includes self-limited acute gastrointestinal infections after consumption of raw or undercooked seafood, wound and soft tissue infections, ear infections, and rarely, septicemia. The majority of infections occur in patients with underlying illnesses (e.g., liver disease, chronic renal failure, diabetes mellitus, and thalassemia major), but also healthy individuals are affected. While almost 80 *Vibrio* species have been identified, currently 12 of them are potential human pathogens (Baker-Austin et al., 2018). Globally, four *Vibrio* species (*V. cholerae*, *V. vulnificus, V. alginolyticus, and V. parahaemolyticus*) are dominant in human infections (Baker-Austin et al., 2017).

In Europe, infections by non-cholera *Vibrio* species are rare, however, a rapidly warming marine environment seems to be an important driver for the emergence and proliferation of *Vibrio* species in the coastal water of the Baltic Sea and North Sea, as *Vibrio* species multiply particularly strongly in warm water above 20°C (Kirschner et al., 2008; Baker-Austin et al., 2017; Marinello et al., 2017; Brehm et al., 2021). The effects of global warming, in particular an increase in the sea surface temperature, have so far been greater in the Baltic Sea than other coastal seas (Belkin, 2009; Markus Meier et al., 2021). A warming trend in the sea surface temperature contributes to the spread of *Vibrio* species in marine environments and will lead to an increase in the number of *Vibrio* infections in vulnerable populations (Vezzulli et al., 2016). However, an increase of *Vibrio* species abundance is not limited to marine and coastal ecosystems only; inland fresh water and natural and artificial lakes can also be affected (Kirschner et al., 2008; Kokashvili et al., 2015; Rehm et al., 2023).

In recent decades, resistance has evolved in several *Vibrio* species toward various antibiotics commonly used for the treatment of infections, such as ampicillin, chloramphenicol, tetracycline, streptomycin, kanamycin, trimethoprim, and carbapenems (Bier et al., 2015). Non-toxigenic *Vibrio* species may act as an environmental reservoir for antibiotic resistance genes (ARGs) which can be acquired through different mechanisms. Chromosomal mutations may contribute to antimicrobial resistance, but acquisition of mobile genetic elements (e.g., plasmids, integrons from closely related strains or other bacterial species) is a key mechanism in multidrug-resistant (MDR) *Vibrio* species (Verma et al., 2019). Acquisition of the gene cassette in Class 1 integrons through horizonal gene transfer has been previously reported in *V. cholerae* isolates derived from stool and rectal swabs in South Africa (Dalsgaard et al., 2001). Comparative genomic analyses of MDR and extensively drug-resistant *V. cholerae* strains isolated from diarrheal samples in India showed that resistance genes are linked to mobile genetic elements and can be easily transferred to other bacterial species (Verma et al., 2019). Another study performed by Borgeaud et al. (2015) on *V. cholerae* O1 El Tor strains revealed that the virulence genes associated with the type VI secretion system, which is a part of the competence regulation, are involved in DNA uptake by bacterial competitors leading to acquisition of antibiotic resistance or virulence genes. *Vibrio* species are naturally present in aquatic environments and can contribute to ARG transfer to humans either through direct contact or via food chains. Therefore, in the context of “One Health,” systematically monitoring the aquatic environment and enhancing the surveillance to identify *Vibrio* species and their antimicrobial resistance is desirable in order to minimize the risk for the human population.

Core genome multilocus sequence typing (cgMLST) is widely used to assess genetic relatedness among bacterial isolates to support outbreak investigation (Pearce et al., 2018) or tracing back transmission chains (Frentrup et al., 2020). The usability of this tool has been reported in comparative genome analysis of *Vibrio parahaemolyticus* in China (Pang et al., 2020) and North America (Xu et al., 2017). Recently, two cgMLST schemes for *Vibrio cholerae* have been developed (Liang et al., 2020; Chen et al., 2022) to facilitate the global epidemiology of cholera disease.

*Vibrio* infections are not always reported across Europe and elsewhere, as they are not classified as notifiable pathogenic agents. This lack of epidemiological data is problematic in the assessment of the overall clinical impact of these infections. In Germany, *Vibrio* species other than toxigenic *V*. *cholerae* strains were not classified as notifiable pathogens before March 2020 (Brehm et al., 2021). Currently, *Vibrio* infections are reported electronically by clinical laboratories to the Robert Koch Institute (RKI) in Germany. We conducted a study to describe virulence and resistance patterns of *Vibrio cholerae* non-O1/non-O139 strains isolated in Germany and other European countries. We also disclosed the genetic relatedness between clinical and environmental *Vibrio cholerae* non-O1/non-O139 isolates using two core genome MLST schemes.

## 2 Materials and methods

### 2.1 Study design and strain collection

We conducted a retrospective study on 87 *V. cholerae* non-O1/non-O139 isolates from clinical (*n* = 63) and environmental (*n* = 24) samples, collected from 2011 until 2021. Although non-cholera *Vibrio* infections were not notifiable in Germany before March 2020, clinical or environmental isolates of *Vibrio cholerae* and other *Vibrio* species could be sent to the Department of Highly Pathogenic Microorganisms (ZBS 2) at the Center for Biological Threats and Special Pathogens of the RKI or to the Federal Institute for risk assessment (BfR) in Germany to confirm the diagnosis and for subtyping. This was voluntarily done by several hospitals and diagnostic laboratories in Germany. Therefore, the RKI holds an extensive bacterial strain collection of *Vibrio* species, which is available for further analyses. We included only *V. cholerae* non-O1/non-O139 isolates for which it was certain that an infection had occurred within Germany or other European countries in this study. We excluded toxigenic *V. cholerae* O1/O139. In total, we examined 63 clinical strains, which had resulted in various manifestations of the disease, including gastroenteritis, wound infections, ear infections, or septicemia. We also included 24 environmental isolates derived from various water sources across Germany, different fish species, and an isolate from a dog with diarrhea. Details of the strain characteristics are given in Table 1 and Supplementary Table 1. For each isolate, information about the source and location of isolation (city) was collected, and when known, data on clinical manifestation and region of potential infection (e.g., travel abroad) were added.

**TABLE 1:** The source of *Vibrio cholerae* non-O1/non-O139 isolates included in the study isolated from environmental and clinical samples from 2011 to 2021.

Source (*n* = 87)			
**Environmental (*n* = 24)**	** No **	**Clinical (*n* = 63)**	** No **
Four-eyed fish (*Anaplebs*)	1	Appendix	1
Fish from fish spa (*Garra rufa*)	6	Blood	10
Hygiene sample (filling line)	2	Ear	31
Stool (dog)	1	Knee (puncture articular fistula)	1
Surface water, bathing site	13	Stool	9
Hygiene sample (water cooling tower)	1	Wound*	11

*Abdomen, leg, shoulder.

*V. cholerae* non-O1/non-O139 isolates from German patients isolated in 2018 and 2019 that were already characterized in the framework of another study (Brehm et al., 2021) were also excluded. Bacterial strains were previously isolated from hospital and clinical laboratories in Germany and Greece. To confirm the identification of *V. cholerae* non-O1/non-O139 sent to RKI for further analyses, and to differentiate *V. cholerae* non-O1/non-O139 from closely related *Vibrionaceae* such as *Vibrio parahaemolyticus* and *Vibrio vulnificus*, two independent real-time multiplex PCR assays were performed. The first real-time multiplex PCR (5′ nuclease assay) detected the *V. cholerae*-specific sequence of the housekeeping gene *sodB* (superoxide dismutase) and also confirmed the lack of enterotoxin gene *ctxA* described previously in details (Dupke et al., 2016). The second real-time multiplex PCR was used for the determination of serogroup-specific gene *rfb* of non-O1/non-O139 *V. cholerae* strains. This PCR was performed to verify that the isolates were not *V. cholerae* O1 or O139 strains that had lost the phage-encoded *ctx* gene before. Primers and probes are described in Supplementary Table 2. PCR conditions were as follows: 50°C for 2 min, 95°C for 10 min, followed by 35 cycles of PCR amplification at 95°C for 15 sec, 55°C for 60 sec.

### 2.2 Antimicrobial susceptibility testing

*Vibrio cholerae* non-O1/non-O139 isolates were cultivated on Columbia Sheep Blood agar (CBA, Oxoid, GmbH, Wesel, Germany), and on a thiosulfate–citrate–bile salts–sucrose agar (TCBS, Oxoid) at 37°C overnight. Antimicrobial susceptibility testing (AST) was performed using the disc diffusion and the gradient strip method for doxycycline only with results categorized applying EUCAST (European Committee on Antimicrobial Susceptibility Testing) breakpoints (version 13.0).^1^ For each bacterial isolate, several morphologically similar colonies were resuspended in 5 ml 0.85% NaCl with a density 0.5 McFarland, streaked onto Muller Hinton agar with tested antibiotics discs/strip and incubated at 36 ± 1°C for 18 ± 2 h. The testing was performed on the following antibiotic discs (Oxoid): piperacillin-tazobactam (36 μg), cefotaxime (5 μg), ceftazidime (10 μg), meropenem (10 μg), ciprofloxacin (5 μg), levofloxacin (5 μg), azithromycin (15 μg), erythromycin (15 μg), tetracycline (30 μg), trimethoprim-sulfamethoxazole (25 μg), and doxycycline [e-test, Liofilchem, Roseto degli Abruzzi (TE), Italy]. *Escherichia coli* ATCC 25922 and *Staphylococcus aureus* ATCC 29213 were used as quality control strains.

### 2.3 DNA extraction and whole genome sequencing

Bacterial DNA was extracted from colony material obtained from overnight cultures on CBA (Oxoid, GmbH, Wesel, Germany) using the Master Pure™ DNA purification Kit (Lucigen, Biozym Scientific GmbH, Hessisch Oldendorf, Germany) following the manufacturer’s instructions. The DNA concentration was assessed on the Qubit^®^2.0 Fluorometer (Life Technologies, Paisley, UK) using the Qubit dsDNA High Sensitivity (HS) assay kit (Life Technologies, Paisley, UK). For all samples, the DNA concentration ranged from 1.27 to 112 ng/μl. The Nextera XT DNA Sample Preparation Kit (Illumina, San Diego, CA, USA) was used for the library preparation, and the sequencing in paired-end mode (2 × 150 bp) was conducted on the NextSeq and MiSeq instrument (Illumina, San Diego, CA, USA). Prior to genome assembly, raw reads were quality controlled using FastQC (Trivedi et al., 2014); Trimmomatics (Bolger et al., 2014) was used for trimming, cropping and adapter clipping. The reads were down-sampled *de novo* assembled with SPADES (v3.15.5) (Bankevich et al., 2012). Also, the CLC Main Workbench (Qiagen, Hilden, Germany) was used for genome assembly. All genome sequences were uploaded to the NCBI Short Sequence Read Archive (SRA).^2^ The BioProject submission ID: PRJNA991120.

### 2.4 cgMLST target scheme definition

The publicly available, finished genome of *V. cholerae* O1 biovar El Tor (NCBI Accession: NC_002505.1, NC_002506.1) was selected as a “seed genome.” To determine the cgMLST gene set, a genome-wide gene-by-gene comparison using the cgMLST Target Definer (v. 1.4) function of the SeqSphere + software (v.7.2.3, Ridom GmbH, Münster, Germany) was performed with set parameters as previously described (Appelt et al., 2022). The identified genes were included in a pairwise comparison using BLAST (v.2.2.12) with parameters as follows: word size, 11; mismatch penalty, −1; match reward, 1; gap open costs, 5; gap extension costs, 2 in order to query chromosomes of selected *V. cholerae* strains. The selected query genomes are publicly available and comprise 13 genomes of *V. cholerae* strains from various outbreaks with different molecular profiles to cover the entire genetic diversity of *V. cholerae* (Supplementary Table 3).

The final cgMLST scheme was formed by including all genes of the reference genome that were common in all query genomes (Supplementary Table 4) with a sequence identity of ≥90% and 100% of overlap. Genes that lack a start or stop codon in one of the query genomes, as well as genes that had internal stop codons in more than 20% of the query genomes, were discarded. The final cgMLST scheme consisted of 2,710 target genes (68.4% of the reference genome *V. cholerae* O1 biovar El Tor str. N16961). The target genes were present in 81.8–97.4% of the examined *Vibrio* species isolates. The cgMLST assay will be made accessible to the public on www.cgmlst.org as soon as the manuscript is published.

### 2.5 cgMLST-based analysis

The genetic relationships of 87 *V. cholerae* non-O1/non-O139 isolates were evaluated by comparing two different cgMLST schemes: (i) the *ad hoc* cgMLST developed in this study (2,710 loci) and (ii) the publicly available *V. cholerae* cgMLST [2,457 defined loci (2,317 core loci)] (Liang et al., 2020); (pubMLST^3^). The Genome Comparator plug-in tool on pubMLST (Jolley and Maiden, 2010) was furthermore used to compare whole genomes of the *V. cholerae* isolates. For phylogenetic reconstructions minimum spanning trees (MST) analysis were performed and missing values were ignored during pairwise distance calculations. The allelic profiles were visualized with GrapeTree with the MSTree V2 algorithm (Zhou et al., 2018).

### 2.6 Virulence and genotypic profiles

The virulence and resistance profiles were determined with functions available on the Ridom SeqSphere + platform. To assess virulence genes, the assemblies of *Vibrio* isolates were compared to sequences stored in the Virulence Factor Database (VFDB) (Chen et al., 2016) with a threshold of >90% sequence identity and 100% coverage. The virulence profiles were then compared to five toxigenic *V. cholerae* O1 or O139 strains: Vib1 (*V. cholerae* NIH41, O1 classic) (Schirmeister et al., 2014), Vib3 (*V. cholerae* HK51, O1 El Tor) (Beutin et al., 1984), Vib4 (*V. cholerae* 1360, O1 classic) (Gallut and Quiniou, 1970), Vib10 (*V. cholerae* A-171-2, O1 El Tor) and Vib28 (*V. cholerae* 186-9, O139 El Tor) (Supplementary Table 1) (SRA BioProject submission ID: PRJNA991120).

The potential *in silico* antibiotic resistance of bacterial isolates was evaluated using the tool AMRfinder [NCBI AMRfinderPlus v2021-06-01.1; (Feldgarden et al., 2019)] focusing on core AMR proteins. The threshold was set to >90% sequence identity and 100% coverage. For visualization of results, the R software (version 4.1.3) was used (Wickham, 2011).

## 3 Results

### 3.1 Antibiotic resistance: antimicrobial susceptibility profile

The summary of the AST results is presented in Table 2. Further details are given in Supplementary Table 5. All of the tested antibiotics are recommended by the EUCAST guideline (v.13.0) for *Vibrio* species. In general, the majority of the examined *V. cholerae* non-O1/non-O139 strains (*n* = 87) were susceptible to beta-lactams (piperacillin-tazobactam, 3rd generation cephalosporin-cefotaxime and ceftazidime, meropenem), quinolones (ciprofloxacin, levofloxacin), macrolides (azithromycin, erythromycin), tetracyclines (tetracycline, doxycycline), and antifolate (trimethoprim-sulfamethoxazole) (Table 2).

**TABLE 2:** Antimicrobial susceptibility testing (AST) and associated antimicrobial resistance determinants found by Illumina sequencing from clinical and environmental *Vibrio cholerae* non-O1/O139 isolates (*n* = 87) from Germany and other European countries, isolated from 2011 to 2021.

Antimicrobial category	Antibiotics	Antibiotic resistance Phenotypic profile							Antibiotic resistance Genotypic profile		
		**Total (*n* = 87)**		**Clinical *n* = 63)**		**Environmental (*n* = 24)**		**Gene** **found**	**Total** **(*n* = 87)**	**Clinical** **(*n* = 63)**	**Environmental** **(*n* = 24)**
		***R***	***S***	***R***	***S***	***R***	***S***		**Positive**	**Positive**	**Positive**
Beta-lactams	TZP	2 (2%)	85 (98%)	–	63 (100%)	2 (8%)	22 (92%)				
	AMP*	–	–	–	–	–	–	*bla*_*CARB–7*_	3	3	–
								*bla*_*CARB–9*_	3	3	–
								*bla*_*PSE*_	4	2	2
	CTX	1 (1%)	86 (99%)	1	62 (99%)	–	24 (100%)	*bla*_*OXA–10*_	3	–	3
	CAZ	1 (1%)	86 (99%)	1 (1%)	62 (99%)	–	24 (100%)				
	MEM	10 (11%)	77 (89%)	–	63 (100%)	10 (42%)	14 (58%)	*varG*	29	26	3
								*bla*_*VCC–1*_	10	–	10
								*bla2/varG*	1	1	–
Quinolone	CIP	–	87 (100%)	–	63 (100%)	–	24 (100%)	*qnrA1* *qnrVC* *qnrVC4* *qnrVC9*	4 1 1 1	– 1 1 1	4 – – –
	LEV	–	87 (100%)	–	63 (100%)	–	24 (100%)				
Macrolide	AZM	–	87 (100%)	–	63 (100%)	–	24 (100%)	*mphF* *arr-2*	3 3	– –	3 3
	E	–	87 (100%)	–	63 (100%)	–	24 (100%)				
	RIF*	–	–	–	–	–	–				
Tetracycline	TET	5 (6%)	82 (94%)	1 (1%)	62 (99%)	4 (17%)	20 (83%)	*tetA* *tetB* tetH**	3 1 1	– – 1	3 1 –
	DOX	5 (6%)	82 (94%)	1 (1%)	62 (99%)	4 (17%)	20 (83%)				
Antifolate	SXT	5 (6%)	82 (94%)	2 (3%)	61 (97%)	3 (13%)	21 (87%)	*dfrA1*	1	1	–
								*dfrA14*	3	–	3
Aminoglycoside	STR*	–	–	–	–	–	–	*aadA2*	1	1	–
								*aadA3*	1	–	1
								*aadA1/* *aadA2*	1	1	–
								*aadA1/* *aadA3*	3	–	3
Fosfomycin	FOS*	–	–	–	–	–	–	*fosB*	1	1	–
Phenicol	C*	–	–	–	–	–	–	*catA2*	1	–	1
								*catA2/cmlA5*	3	–	3
								*catB9*	20	19	1
Sulfonamide	SUL*	–	–	–	–	–	–	*sul1*	3	2	1
								*sul1/sul2*	3	–	3

S, susceptible; R, resistant based on the EUCAST criteria; TZP, piperacillin-tazobactam; AMP, ampicillin; CTX, cefotaxime; CAZ, ceftazidime; MEM, meropenem; CIP, ciprofloxacin; LEV, levofloxacin; AZM, azithromycin; E, erythromycin; RIF, rifamycin; TET, tetracycline; DOX, doxycycline; SXT, trimethoprim-sulfamethoxazole; STR, streptomycin; FOS, fosfomycin; C, chloramphenicol; SUL, sulfonamide; *antibiotics not tested, ** gene identified by AMPfinder below applied threshold (identity: 90%, coverage: 100%). AST performed by disc diffusion method for all antibiotics except doxycycline (gradient strip).

Based on the AST, 10 out of 24 environmental isolates (42%) were resistant to meropenem, two (8%) environmental isolates were resistant to piperacillin-tazobactam, and one clinical isolate was resistant to cefotaxime and ceftazidime. Five isolates (one clinical and four environmental) were resistant to tetracycline and doxycycline, and two clinical and three environmental isolates were resistant to trimethoprim. All strains were susceptible to ciprofloxacin, levofloxacin, azithromycin and erythromycin. Based on the definition of multidrug resistance (acquired non-susceptibility to at least one agent in three or more antimicrobial categories) (Magiorakos et al., 2012), we detected two environmental isolates out of all examined samples that were resistant to three antibiotic classes (beta-lactam: piperacillin-tazobactam; tetracyclines: tetracycline, doxycycline; and antifolate: trimethoprim-sulfamethoxazole).

### 3.2 Antibiotic resistance: genotypic profile

**FIGURE 1:**
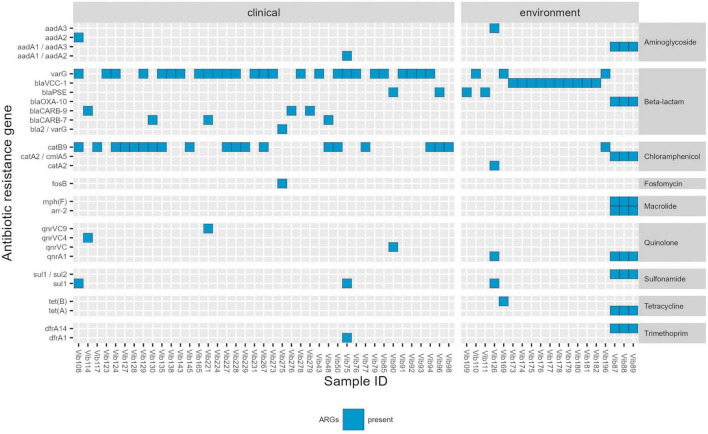
Genotypic profile of *Vibrio cholerae* non-O1/non-O139 isolates (*n* = 60) isolated in Germany and other European countries from 2011 to 2021. Legend: ARGs-Antimicrobial resistance genes. Threshold of >90% sequence identity and 100% of coverage against targets was applied.

Detailed information regarding the genotypic profiles of *V. cholerae* non-O1/non-O139 isolates are given in Table 2 and Supplementary Table 6. The genotypic profiles disclosed antibiotic resistance genes (ARGs) that confer resistance to beta-lactams, quinolones, macrolides, tetracycline, antifolate, aminoglycosides, fosfomycin, phenicols and sulfonamide (Table 2; Figure 1). Among 87 isolates the sequencing revealed the presence of ARGs in 60 *Vibrio* isolates examined. Those included beta-lactamase genes belonging to Class A beta-lactamases: *bla_*CARB–*7_* (*n* = 3), *bla_*CARB–*9_* (*n* = 3), and *bla*_*PSE*_ (*n* = 4); Class D beta-lactamases: *bla_*OXA–*10_* (*n* = 3), and metalo-beta-lactamases: *varG* (*n* = 29), *bla_*VCC–*1_* (*n* = 10) and one *bla2/varG*. *Qnr* resistance determinants are associated with low-level resistance or reduced susceptibility to quinolone. Here, despite the detection of several *qnr* resistance genes, all isolates were phenotypically susceptible to this agent, which could be associated with poor expression, silencing or inactivation of these genes. Genes that encode for resistance to macrolides (*mphF*) were identified in three environmental isolates, although no erythromycin resistance was observed in these isolates. Tetracycline (*tetA/tetB*/*tetH*) genes were detected in all five isolates with phenotypic tetracycline and doxycycline resistance. Trimethoprim (*dfrA1/dfrA14*) determinants were identified in four out of five isolates also phenotypically resistant to trimethoprim. In this study we only focus on antibiotics that are recommended by EUCAST for the treatment of *Vibrio* species infections, therefore AST was not performed on aminoglycosides, fosfomycin, rifamycin, chloramphenicol and sulfonamide, however, several associated ARGs were found.

### 3.3 Virulence genes profile

**FIGURE 2:**
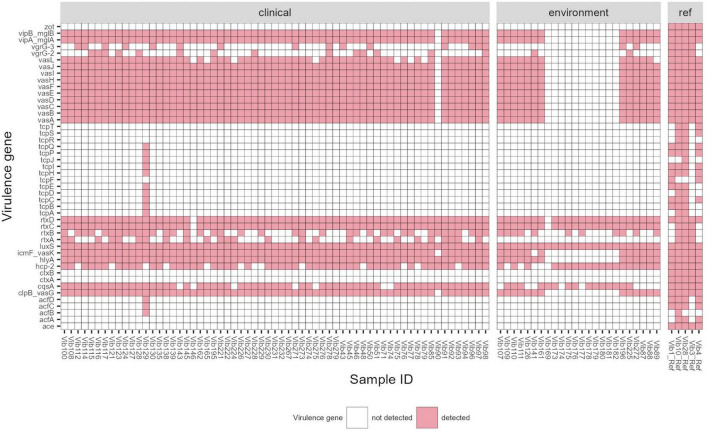
Virulence gene profile of *Vibrio cholerae* non-O1/non-O139 isolates (*n* = 87) isolated in Germany and other European countries from 2011 to 2021. Reference strains used for comparison: Vib1 (*V. cholerae* NIH41, O1 classic), Vib3 (*V. cholerae* HK51, O1 El Tor), Vib4 (*V. cholerae* 1360, O1 classic), Vib10 (*V. cholerae* A-171-2, O1 El Tor) and Vib28 (*V. cholerae* 186-9, O139 El Tor). Threshold of >90% sequence identity and 100% of coverage against targets was applied.

Among 87 *Vibrio* isolates, whole genome sequencing identified 37 virulence genes (VGs). The range of VGs per bacterial isolate was 2–34 with an average of 18 VGs per isolate. In total 38 virulence profiles were observed among all clinical and environmental isolates (Figure 2; Supplementary Table 7). None of the virulence profiles dominated in tested strains, however, one profile was observed more frequently, and it was detected in 8 clinical strains isolated from various sites (5 from ear, 2 from wound and one from blood culture) between 2014 and 2021. This virulence profile comprised the following genes: *clpB, cqsA, hcp-2, hly-A, icmF, luxS, rtxC-D, vasA-L, vipA/mglA* and *vipB/mglB*. Generally, virulence profiles were similar between clinical and environmental isolates, except for one clinical-stool isolate (Vib129) with the highest number (34 out of 37) of VGs detected and a cluster of 11 environmental isolates (Vib169, Vib173 to Vib182) with the lowest number of VGs found. The former isolate-Vib129, acquired in Netherlands during a cruise in 2016, possessed additional accessory colonization factors *acfB/C/D* and a cluster of toxin-coregulated pilus genes *tcpA-E/H-J/P/Q* which might be linked to toxigenic *V. cholerae* as possible origin. This isolate could have acquired the cluster of *tcp* proteins from toxigenic *Vibrio* isolates or alternatively remove the CTX element from toxigenic TCP positive *Vibrio* strains. Ten out of the eleven environmental isolates (Vib173-Vib182) with only 4–6 virulence genes were derived from the surface water in North Rhine-Westphalia, Germany (Baldeneysee, dammed river Ruhr) collected in 2018. The remaining Vib169 strain from that cluster, isolated from a fish (*Garra rufa*), contains only two VGs (*cqsA, luxS*). None of the tested isolates contained the cholera toxin genes (*ctxA/ctxB*) and the main virulence factors of the CTX phage such as *zot* (zonula occludens toxin), accessory cholera enterotoxin (*ace*), and factors for phage coat proteins (*orfU*).

The prevalence of virulence factors found in clinical and environmental isolates is listed in Table 3. All examined isolates carried the *luxS* gene which is a part of the *Vibrio* species quorum sensing system controlling the expression of multiple genes in response to population density (Ye et al., 2008). The predominant virulence factors in *V. cholerae* non-O1/non-O139 were repeats-in-toxins- *rtxC/rtxD* (98%) and hemolysin- *hlyA* (87%), followed by 85–86% of isolates possessing different virulence-associated type VI secretion proteins (T6SS)- *vasA-K, clpB, vipA/mglA, vipB/mglB*, *icmF.* Other common virulence factors were hemolysin-coregulated protein- *hcp-2* and quorum sensing protein- *cqsA* (79 and 82%, respectively). Other, less frequently found virulence proteins included *rtxA* (31%) and *rtxB* (56%) and proteins associated with T6SS–*vgrG-2* (18%) and *vgrG-3* (20%). *rtxA* gene was identified only in clinical isolates (*n* = 25), and in 7 (28%) out of these 25 isolates were from stool isolates.

**TABLE 3:** Frequency of virulence factors in clinical and environmental *Vibrio cholerae* non-O1/non-O139 isolates isolated in Germany and other European countries from 2011 to 2021.

Name	Virulence genes (*n* = 37)	Total no of strains (*n* = 87; 100%)	No of clinical isolate (*n* = 63)	No of environmental isolate (*n* = 24)	No of reference strains (*n* = 5)
Vibrio pathogenicity island 1 (VPI-1)/toxin-coregulated pilus Accessory virulence factors	acfA*	–	–	–	3
	*acfB*	1	1	–	1
	*acfC*	1	1	–	4
	*acfD*	1	1	–	4
Type 6 secretion system (T6SS) core genes	*clpB/vasG*	75 (86%)	62	13	5
Quorum sensing/Autoinducer synthase	*cqsA*	71 (82%)	56	15	5
Hemolysin	*hcp-2*	69 (79%)	50	19	5
Cytotoxin/Hemolysin	*hlyA*	76 (87%)	63	13	5
Inner membrane protein vasK	*icmF/vasK*	74 (85%)	62	12	5
S-ribosylhomocysteinase Quorum sensing/autoinducer	*luxS*	87 (100%)	63	24	5
Multifunctional autoprocessing repeat-in-toxins	*rtxA*	25 (31%)	25	–	3
	*rtxB*	49 (56%)	38	11	3
	*rtxC*	85 (98%)	62	23	3
	*rtxD*	85 (98%)	62	23	5
Vibrio pathogenicity island 1 (VPI-1)/toxin-coregulated pilus	*tcpA*	1	1	–	2
	*tcpB*	1	1	–	3
	*tcpC*	1	1	–	4
	*tcpD*	1	1	–	2
	*tcpE*	1	1	–	3
	tcpF*	–	–	–	2
	*tcpH*	1	1	–	4
	*tcpI*	1	1	–	4
	*tcpJ*	1	1	–	1
	*tcpP*	1	1	–	4
	*tcpQ*	1	1	–	4
	tcpR*	–	–	–	2
	tcpS*	–	–	–	3
	tcpT*	–	–	–	3
Type 6 secretion system (T6SS) core genes	*vasA*	75 (86%)	62	13	5
	*vasB*	75 (86%)	62	13	5
	*vasC*	75 (86%)	62	13	5
	*vasD*	75 (86%)	62	13	5
	*vasE*	75 (86%)	62	13	5
	*vasF*	75 (86%)	62	13	5
	*vasH*	75 (86%)	62	13	5
	*vasI*	75 (86%)	62	13	5
	*vasJ*	75 (86%)	62	13	5
	*vasL*	67 (77%)	55	12	5
Type 6 secretion system (T6SS) core genes	*vgrG-2*	16 (18%)	14	2	4
	*vgrG-3*	17 (20%)	15	2	4
Type 6 secretion system (T6SS) core genes	*vipA/mglA*	75 (86%)	62	13	5
	*vipB/mglB*	75 (86%)	62	13	5
Zonula occludens toxin	zot*	–	–	–	5
CTX prophage/Cholera toxin	ctxA*	–	–	–	5
	ctxB*	–	–	–	5
Accessory cholera enterotoxin (Membrane-acting toxin)	ace*	–	–	–	5

*Virulence genes found only in reference strains.

### 3.4 Genetic relatedness between the isolates using cgMLST schemes

**FIGURE 3:**
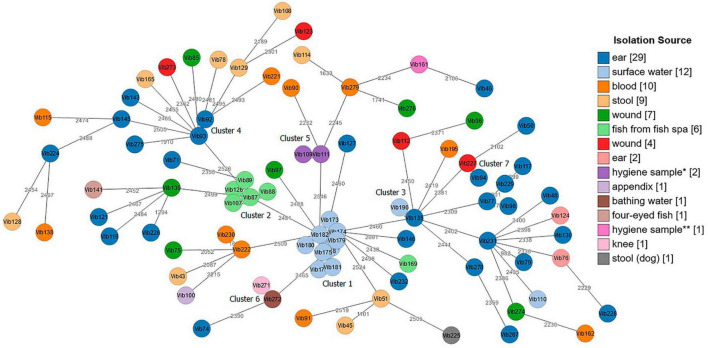
Minimum spanning tree (MST) of *Vibrio cholerae* non-O1/non-O139 isolates (*n* = 87) generated from *ad hoc* cgMLST Ridom SeqSphere + scheme. Distance based on 2,710 core genes *V. cholerae* N16961 reference strain. Each circle represents one isolate. Numbers between nodes indicate allelic distances between the isolates (maximum allelic distance 2,450). MST cluster distance threshold: 20. Isolates are color-coded based on their isolation source. The visualization of the tree was performed using stand-alone GrapeTree software. *Hygiene sample (filling line); **hygiene sample (water cooling tower).

**FIGURE 4:**
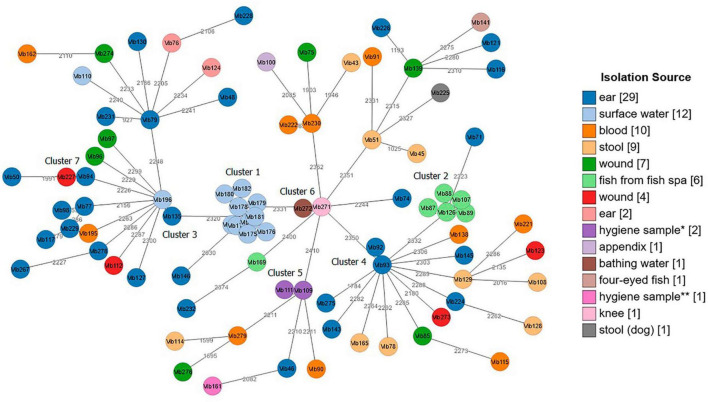
Minimum spanning tree (MST) of *Vibrio cholerae* non-O1/non-O139 isolates (*n* = 87) generated from *Vibrio cholerae* pubMLST scheme. Distance based on 2,457 defined loci. Each circle represents one isolate. Numbers between nodes indicate allelic distances between the isolates (maximum allelic distance 2,435). MST cluster distance threshold: 20. Isolates are color-coded based on their isolation source. The visualization of the tree was performed using stand-alone GrapeTree software. *Hygiene sample (filling line); **hygiene sample (water cooling tower).

Both tested schemes were comparable and revealed identical groupings of closely related isolates with only few allele differences between the two assays. The minimum spanning tree (MST) generated from the *ad hoc* cgMLST scheme using the Ridom SeqSphere + software is shown in Figure 3. It displayed a high level of genetic diversity between epidemiologically unlinked *V. cholerae* non-O1/non-O139 isolates with the maximum allelic distances of 2,450 (Supplementary Table 8). The minimum spanning tree (MST) generated from publicly available *Vibrio cholerae* cgMLST scheme on the pubMLST is presented in Figure 4. The maximum allelic distance among isolates was 2,435 and the distance matrix is shown in Supplementary Table 9. The allelic profiles for *ad hoc* cgMLST scheme (Ridom SeqSphere +) and pubMLST scheme are given in Supplementary Table 10 and Supplementary Table 11, respectively.

Both cgMLST assays identified 7 clusters with closely related *Vibrio* isolates (allelic distances < 20) suggesting a possible epidemiological linkage (Figures 3, 4). Three out of these 7 clusters (Cluster 1, 2, and 5) derived from various environmental sources. Cluster 4 and Cluster 7 contained samples isolated from clinical sites, and Clusters 3 and 6 included samples collected from both clinical and environmental sources. Cluster 1, consisting of strains Vib173-Vib182 comprised the largest number (*n* = 10) of closely related isolates with less than 5 (Ridom) and less than 20 (pubMLST) allelic distances. These isolates were collected within 2 days from the same surface (fresh) water (Baldeney See, dammed river Ruhr) in North Rhine-Westphalia, within the context of the 2018 bathing water quality project in Germany (Schmithausen et al., 2019).

Cluster 2, the second largest cluster, contained 5 isolates (Vib87-Vib89 and Vib107, Vib126) with allelic distances less than 10 (Ridom) and less than 20 (pubMLST). Swabs from isolates of this cluster were taken from the mucus membrane of fish (*Garra rufa*) from a fish spa at different collection dates. Three out of these 5 isolates (Vib87-Vib89) derived from the same sample (2014), but isolates differed phenotypically. Two other isolates (Vib107 and Vib126) were collected from fish of two different spa-pools of the same location, and were collected in 2015 and 2016, respectively. The close genetic relationship of all isolates from Cluster 2 indicates the long-term presence of a single strain in both spa-pools. Another isolate from this spa (Vib169), isolated in 2018, differed in more than 2,000 alleles, which suggests the presence of a second, distantly-related strain at this location.

The remaining five clusters consisted of two isolates each. Cluster 3 comprised strains Vib135 and Vib196. While isolate Vib135 was taken from the ear of a patient with chronic otitis in 2017, isolate Vib196, with a difference of six alleles (Ridom) and 11 alleles (pubMLST), was isolated in 2019 from an environmental source (bathing water). A possible epidemiological linkage exists insofar as the patient lived in close proximity to the bathing area. However, it was not possible to determine whether the patient actually bathed in the water. On the contrary, strain Vib195, isolated from blood culture from another patient who had been shown to have bathed in the same bathing water shortly before the sample from which strain Vib196 was isolated, showed a difference of over 2,000 alleles. Despite the spatial proximity and the temporal connection, an epidemiological linkage could not be confirmed in this case.

Cluster 4 included two closely related isolates Vib92 and Vib93 (one allele difference in Ridom and 3 alleles difference in pubMLST) collected from the same patient’s ear in 2014. Cluster 5 compromised two almost identical environmental hygiene isolates Vib109 and Vib111 (no allele difference in Ridom and two alleles differences in pubMLST). Both isolates were collected from the filling line in 2015 in Belgium but they differed phenotypically. Cluster 6 comprised two strains (Vib271 and Vib272) that were indistinguishable in cgMLST analysis. Vib271 was isolated from the knee synovia of a patient, whereas strain Vib272 was isolated from the bathing site where the patient had previously bathed. In contrast to Cluster 3, a clear epidemiological connection between the patient isolate and the isolate from the bathing site could be demonstrated in this case. Cluster 7 included two clinical isolates, Vib94 and Vib227, isolated from two patients from ear and wound sites with less than 20 (both schemes) allelic differences.

## 4 Discussion

In Germany, non-cholera *Vibrio* infections have been reported from coastal waters of the Baltic Sea and North Sea (Boer et al., 2013) with seasonal correlation. Most cases are registered in the warmer months and males are affected more often than females (Brehm et al., 2021). In the framework of a German research project, the VibrioNet^4^ the *Vibrio* database (VibrioBase) was created to improve identification of marine environmental *Vibrio* species (Erler et al., 2015) and to investigate vibriosis among clinical sources across Europe. Several studies in Germany revealed the genotypic and phenotypic patterns of non-toxigenic *Vibrio* species among clinical and environmental isolates (Schirmeister et al., 2014; Bier et al., 2015; Hammerl et al., 2017; Schwartz et al., 2019; Brehm et al., 2021). The study published by Hammerl et al. (2017) showed that carbapenemase-producing non-O1/non-O139 *V. cholerae* isolates are present in aquatic environments in Germany and represent environmental reservoir of carbapenem resistance. To get an insight into molecular epidemiology of *Vibrio* species isolates available in Germany, we conducted a retrospective study to disclose virulence and resistance patterns of *Vibrio cholerae* non-O1/non-O139 isolates derived from Germany and other European countries from 2011 to 2021. We also investigated the genetic relatedness between clinical and environmental *V. cholerae* non-O1/non-O139 isolates using two cgMLST schemes.

### 4.1 Antimicrobial susceptibility profile

A recent guideline in Germany recommended tetracycline (doxycycline) and 3rd generation cephalosporin (ceftriaxone) for skin and soft tissue infections associated with exposure to water that is contaminated by *Vibrio* species (Sunderkotter et al., 2019). These antibiotic agents have been also successfully used in the treatment of bacteremia caused by non-toxigenic *Vibrio* species (Marek et al., 2013; Lan et al., 2014). Moreover, treatment with piperacillin/tazobactam (Kadkhoda et al., 2012), azithromycin (Marinello et al., 2017), fluoroquinolone (Chowdhury et al., 2016), ampicillin (Feghali and Adib, 2011), and meropenem (Torp-Pedersen et al., 2012; Lu et al., 2014) has been well described in non-O1/non-O139 *Vibrio cholerae* infections. While researchers pay attention to antimicrobial resistance patterns in toxigenic *Vibrio cholerae*, such data is rarely published for non-toxigenic *Vibrio* isolates.

In our study, the proportion of resistance among clinical and environmental isolates against antimicrobial agents recommended as first line treatment for *Vibrio* infections were low meaning that empirical treatment would likely have been effective. Similar observations have been reported in German studies (Bier et al., 2015; Brehm et al., 2021) and also in other countries (Ceccarelli et al., 2015; Ottaviani et al., 2018). We noted the highest resistance rate among 10 environmental isolates against beta-lactams, specifically meropenem (42%). These 10 out of the 24 tested environmental strains were collected within 2 days from bathing waters (Baldeneysee, dammed river Ruhr) in North Rhine-Westphalia, Germany as part of the screening project to seek antibiotic-resistant intestinal bacteria (*Escherichia coli*, other Coliforms and Enterococci) and antibiotic residues (Schmithausen et al., 2019). The investigation resulted in an unexpected outcome when non-toxigenic *Vibrio* species were detected. Among all examined isolates we reported two environmental MDR non-toxigenic *Vibrio* species isolates that revealed resistance to three antibiotic classes: beta-lactam (piperacillin-tazobactam), tetracyclines (tetracycline, doxycycline) and antifolate (trimethoprim-sulfamethoxazole). These two isolates were collected from the same source (routine testing of *Garra rufa* fish from fish tank). *Garra rufa* is a freshwater fish naturally inhabiting river basins in central Eurasia. They are widely used in cosmetic treatment, in particular for pedicures and body spa to remove dead skin. Identification of *Vibrio* species from this fish species was previously published (Verner-Jeffreys et al., 2012), however, the antimicrobial resistance pattern was not tested. In contrast to our outcome, the study performed by Bier et al. (2015) in Germany on a broad collection of clinical, environmental and sea food, non-O1/O-139 *Vibrio* isolates did not reveal any multidrug-resistant strains. Our finding suggests that MDR *Vibrio* strains are present in environmental sources, and might contribute to transmission of antibiotic resistance from fish or water in a fish spa to vulnerable clients.

### 4.2 Antimicrobial resistance genes profile

Genotypic profiling disclosed antibiotic resistance genes that mediate resistance to several antibiotic classes, however, not all were confirmed by phenotypic profiling. In our study, we detected several beta-lactamase genes both in clinical and environmental isolates. These included carbenicillin-hydrolyzing beta-lactams: *bla_*CARB–*7_, bla_*CARB–*9_* and *bla*_*PSE*_ whose presence has already been described in non-toxigenic *Vibrio* isolates (Lepuschitz et al., 2019; Brehm et al., 2021; Adesiyan et al., 2022) suggesting activity against amino- and carboxy-penicillin (Lepuschitz et al., 2019), but susceptibility testing was not performed on these antibiotic agents. Most antibiotic resistance determinants are encoded by gene cassettes of Class 1 integrons which compromise two conserved segments (5′-CS and 3′-CS) broken down by variable region (Stokes and Hall, 1989). However, both *bla_*CARB–*7_* and*bla_*CARB–*9_* are located in the super integron (VCR island) of the *V. cholerae* genome (Melano et al., 2002; Petroni et al., 2004). Sequence analysis of three environmental isolates (Vib87, Vib88, Vib89), collected from fish (*Garra rufa*), revealed the presence of *bla_*OXA–*10_* and various other resistance determinants that encode for trimethoprim (*dfrA14*) and tetracycline (*tetA*), both confirmed by phenotypic profile; sulfonamide resistance (*sul1, sul2*), quinolone resistance (*qnrA1*), macrolide resistance (*arr-2, mphF*) and chloramphenicol resistance (*cat2, cmlA5*), and aminoglycoside resistance (*aadA1, aadA2*). The MDR profile disclosed in Vib87 and Vib88 isolates might be associated with the presence of a 7.5 kb plasmid and a very low copy number plasmid of unknown molecular size as well as Class 1 integrons as previously described (Rajpara et al., 2009). The study revealed that the mechanism of multidrug-resistance of *Vibrio fluvialis* isolates, acquired from patients with acute cholera-like diarrhea in Kolkata, India, is associated with the acquisition of the 7.5 kb pVN84 plasmid originating from *Vibrio cholerae* O1 in Vietnam indicating the plasmid exchange between two species. The follow up study (Rajpara et al., 2018) on the same MDR *V. fluvialis* isolate showed a similar genetic profile, as described here, located on the low copy number plasmid that bears multiple resistance genes.

The largest number of *Vibrio* isolates exhibited genotypic resistance to *varG* and *bla_*vcc–*1_*, which demonstrated activity against penicillin, cephalosporins, and carbapenems (Mangat et al., 2016; Lin et al., 2017). The high prevalence of the *varG* gene has been demonstrated in a recent study conducted on various *Vibrio* species isolates collected from human, food, animal and environmental sources in Latin America (Vilela and Falcão, 2021). Carbapenemase VCC-1, which produces non-toxigenic *V. cholerae* strains, was described for the first time by Canadian researchers in 2016 (Mangat et al., 2016). *bla_*vcc–*1_* was identified in a shrimp imported into Canada for human consumption. A similar finding was published a year later by a German study (Hammerl et al., 2017). They detected *bla_*vcc–*1_* in several non-toxigenic *V. cholerae* strains collected from different locations on the coastline waters in Germany. Similarly, we detected *bla_*vcc–*1_* in ten meropenem-resistant non-O1/non-O139 *V. cholerae* isolates acquired from surface water in Germany during routine testing. This finding suggested that *V. cholerae* strains which produce carbapenemase VCC-1 are continuously present in German waters and might be acquired by seafood or fish inhabiting the aquatic environments, as it was discovered in Canada, entering the human body and developing various clinical symptoms.

The presence of other as-yet unmentioned genetic determinants that confer resistance to aminoglycoside [*aph(6)-Id*], chloramphenicol (*floR, catB9*), quinolone (*qnrVC*), tetracycline (*tetB*) and trimethoprim (*dfrA1*) has already been described in *Vibrio* species (Rahmani et al., 2012; Lepuschitz et al., 2019; Jackel et al., 2020). The *fosB* gene, which confers resistance to fosfomycin, was identified in one of our clinical isolates collected from an ear location with clinical manifestation as otitis media. Fosfomycin is usually used for the treatment of urinary tract infections caused by *Enterobacterale*s infections (Falagas et al., 2010) and has a moderate activity against *Vibrio* species (Reparaz and Fernandez, 1977). It is not a common agent in the treatment of *Vibrio* infections, although it was used in combination with other antibiotics for the treatment of necrotizing fasciitis of the lower leg caused by non-toxigenic *Vibrio cholerae* (Hirk et al., 2016). To our knowledge, the presence of this determinant in *Vibrio* species has never yet been reported, and no resistance to fosfomycin has been described.

### 4.3 Virulence gene profiles and genetic diversity

Two cgMLST schemes have been used to assess genetic relatedness between clinical and environmental isolates. Both cgMLST schemes yielded comparable results, and disclosed high genetic diversity between all tested isolates. Similar findings have been seen in *V. cholerae* non-O1/non-O139 environmental strains of the Baltic Sea in a German study (Schwartz et al., 2019). Both cgMLST schemes also revealed seven clusters, each compromising closely related isolates with 0–19 (Ridom) and 0–18 (PubMLST) allelic differences. Most of the clusters contained isolates with regional or within-patient pattern, except two isolates in Cluster 7 which derived from different clinical sources and regions. We did not observe a distinct country-wide spread of clonal lineages. For the PubMLST cgMLST assay, a threshold of 7 allelic differences was determined for strains of the same outbreak or with a clear epidemiological linkage (Liang et al., 2020) and up to 40 allelic differences for closely related strains. Based on these cut-offs and by directly comparing the allelic differences of both assays, an approximate cut-off of 3 allelic differences for outbreak strains and a cut-off of 20 for closely related strains can be applied for the *ad hoc* scheme.

Numerous studies aimed to disclose virulence genes in non-toxigenic *Vibrio cholerae* non-O1/non-O139 isolates by PCR (Schirmeister et al., 2014; Schwartz et al., 2019); they mainly focused on well-known targets. Here, we investigated virulence gene profiles by a whole-genome sequencing-based approach which delivered a more comprehensive picture than targeted PCR alone. In total, we identified 38 different virulence profiles among all 87 *Vibrio* isolates. Though we observed genetic diversity among clinical and environmental isolates in general, virulence gene profiles have similar patterns regardless of the source of the isolates. A similar finding was noted in German and Austrian studies (Schwartz et al., 2019; Bhandari et al., 2023), in which virulence factors found in clinical isolates were also present in environmental strains regardless of sequence type. The major virulence factors, *ctxAB, zot, ace*, and *rstA/B/R*, were not detected in the examined isolates, while two clusters of toxin-coregulated pilus genes (*tcpA-E/H-J/P/Q* and *acfB/C/D*) were identified in one clinical isolate recovered from a stool sample. The presence of these two virulence clusters, especially *tcpA* and *acfB* genes, have been reported in both clinical and environmental non-O1/non-O139 *V. cholerae* strains (Novais et al., 1999; Theophilo et al., 2006; Jagadeeshan et al., 2009). The pilus colonization clusters (TCP) are located on the *Vibrio* pathogenicity island (VPI-1) and play a role in attachment to the host epithelial cells. They also serve as a receptor for CTX prophage (Krebs and Taylor, 2011). *Vibrio cholerae* isolates that are negative for *ctxAB* but positive for *tcpA* have the highest risk of disease outbreak among the non-toxigenic *Vibrio* isolates with mild or moderate clinical symptoms (Wang et al., 2020). Some authors suggest that non-toxigenic *Vibrio* strains with a profile *ctxAB ^–^/tcpA^+^* might be a precursor of toxigenic strains due to the function of the TCP element (Theophilo et al., 2006), and such profile has been observed in our study in one clinical isolate acquired from a stool sample.

Human infections with non-toxigenic *V. cholerae* mostly manifest as an acute gastroenteritis, ear infections, or severe wound infections. It is important to identify the virulence factors associated with the pathogenesis of these infections. We looked closer into the virulence profile of non-toxigenic *Vibrio* strains isolated from stool, wound and ear sites. Some of the virulence genes were present in all strains, and some were identified only with a low frequency, e.g., valine-glycine repeat protein G (*vgrG*-2, *vgrG*-3) which plays role in competing against the microbial community in the natural environment or in the human gastrointestinal tract (Brooks et al., 2013). A German study revealed that among environmental and clinical strains of non-cholerae *Vibrio* isolates, the most frequently found virulence genes were *toxR*, El Tor *hlyA, ompU* and *rtxC* (Schwartz et al., 2019). Another study conducted on non-O1/non-O139 *V. cholerae* environmental isolates in Maryland (Ceccarelli et al., 2015) showed the predominance of *hlyA, hap, rtxA, vasA, vasK* and *vasH* genes. Here, we did not observe any *toxR* and *ompU* genes, and we did not differentiate classical *hlyA* gene from El Tor *hlyA* type. All isolates examined from stool, wound and ear sites in this study showed the presence of virulence genes for T6SS (*clpB, vasA-L*, *vipA-B*), quorum sensing genes (*luxS*), hemolysin gene (*hlyA*), inner membrane protein (*icmF*) and repeats-in-toxins genes (*rtxC-D*), which agreed with previous studies conducted in non-cholera *V. cholerae* isolates from ducks (Hirsch et al., 2020), clinical sites (Brehm et al., 2021) and environmental source (Shan et al., 2022).

T6SS contains virulence factors that play a role in survival in both the aquatic and host environments by competitive exclusion of bacterial competitors (MacIntyre et al., 2010), promote intestinal colonization in the host indicating intestinal inflammation *in vivo* (Ma and Mekalanos, 2010) and also foster horizontal gene transfer (Borgeaud et al., 2015). The activity of T6SS is regulated by various mechanisms, such as the expression of hemolysin co-regulated proteins (*hcp-2* and *hcp-*3) controlled by a quorum sensing system (QSS) (Ishikawa et al., 2009). The present of *hcp* genes was observed in more than half of those isolates (6 out of 9 strains from stool, 24 out of 31 strains from ear and 9 out of 11 from wounds). QSS is bacterial monitoring system involving in various functions, e.g., to control population density in the specific niche, biofilm formation and virulence genes expression. Two genes, *luxS* and *cqsA*, are a part of the QSS system, and they were identified in all examined isolates acquired from stool and wound, while *cqsA* was detected in 81% of the isolates collected from ears. These two virulence genes were also found in environmental non-toxigenic *Vibrio* strains (Schwartz et al., 2019).

The presence of the hemolysin gene (*hlyA*), involved in cytotoxicity and apoptosis induction (Kanoktippornchai et al., 2014), and repeats-in-toxin gene cluster (*rtxA-D*) have been widely observed in non-O1/non-O139 *Vibrio* isolates collected from the coastal waters of the Baltic Sea and the North Sea (Schwartz et al., 2019) and in other studies (Dutta et al., 2013; Ceccarelli et al., 2015). Here, *hlyA* and *rtxC-D* proteins were observed in most of the tested isolates, while other repeats-in-toxins genes, *rtxA* and *rtxB*, were less common. The repeats-in-toxin RTX is a group of cytolysins and cytotoxins produced by gram-negative bacteria. RTX proteins are defined by the presence of characteristic repetitions of glycine and aspartate in the toxin protein sequence and by the unique mode of the secretion type I extracellular secretion system (TISS) (Linhartova et al., 2010). A study conducted by Schirmeister et al. (2014) revealed that the *rtxA* gene occurred only in non-toxigenic *Vibrio* recovered from diarrhea samples. Similar to our study, the *rtxA* gene was only identified in clinical isolates (Table 3) and of those, *rtxA* was observed in 7 out of 9 (77%) clinical stool isolates. To get an insight into those isolates we checked the metadata, however, they turned out to be incomplete. Three patients reported travelling abroad (Greece, Amsterdam and Danube Delta), and in one patient, consumption of crabs in a German city was reported as causing an infection. *rtxA* encodes multifunctional autoprocessing repeats-in-toxin, and while its role it not entirely clear, it is suspected that it is linked to increased epithelial cell damage in the *in vivo* model (Satchell, 2007; Lee et al., 2008).

## 5 Conclusion

The main aim of this study was to perform molecular characterization of non-O1/non-O139 *Vibrio cholerae* isolates isolated from environmental and clinical samples, including the determination of virulence and antibiotic resistance gene patterns, and to assess whether these isolates were closely genetically related. We found that most of the isolates were susceptible to the antibiotics we tested and recommend that first line treatment for *Vibrio* infection would likely be effective in clinical isolates if needed. Concern remains over the escalating carbapenemase VCC-1 produced by non-toxigenic *V. cholerae* strains in aquatic environments. The results of this analysis suggested that carbapenemase-producing non-O1/non-O139 *V. cholerae* are constantly present in surface water in Germany, and that multidrug-resistant *Vibrio* isolates are also found in fish spas. Nowadays non-cholera *Vibrio* species are frequently detected in aquatic environments in Europe, and due to climate change, the abundance of *Vibrio* species in the aquatic niche might rise; this may lead to an increase in *Vibrio* species infections in the human population. The virulence profile disclosed that both clinical and environmental isolates possess similar virulence factors. The main virulence genes originally presence in toxigenic *Vibrio cholerae* were not common in examined isolates, however, some, like the cluster of toxin-coregulated pilus genes, can be sporadically isolated in clinical isolates manifesting with gastrointestinal symptoms. Due to horizontal virulence and resistance gene transfer, systematic monitoring in aquatic environments should be considered as one of the counter-measures to seek antimicrobial resistance and virulence patterns among *Vibrio* isolates. The genetic diversity among our isolates was high, however, we identified a cluster with identical environmental and clinical isolates, which suggests that direct exposure of water that has been contaminated by *Vibrio* species may be one of the sources of acquiring the infection. More studies are needed to truly understand the molecular basis of genetic elements involved in pathogenicity of *V. cholerae* non-O1/non-O139 and how they are transmitted.

## Data availability statement

The original contributions presented in the study are publicly available. This data can be found here: https://www.ncbi.nlm.nih.gov/bioproject/; PRJNA991120/.

## Author contributions

KS: Conceptualization, Formal analysis, Investigation, Methodology, Validation, Writing—original draft, Writing—review and editing, Data curation, Visualization. HS: Conceptualization, Data curation, Formal analysis, Investigation, Methodology, Validation, Writing—original draft, Writing—review and editing, Resources. SA: Data curation, Methodology, Validation, Writing—review and editing. JM: Writing—review and editing, Formal analysis, Investigation. DJ: Formal analysis, Writing—review and editing, Conceptualization, Methodology, Resources, Supervision, Validation, Writing—original draft. SD: Conceptualization, Formal analysis, Methodology, Resources, Supervision, Validation, Writing—original draft, Writing—review and editing, Investigation, Project administration.

## Footnotes

1
www.eucast.org

2
www.ncbi.nlm.nih.gov/sra

3
https://pubmlst.org/vcholerae/

4
http://www.vibrionet.de/
