# Supplementary material for: Virulence and resistance patterns of Vibrio cholerae non-O1/non-O139 acquired in Germany and other European countries
Source: Front Microbiol. 2023 Nov 22;14:1282135. doi: 10.3389/fmicb.2023.1282135 (PMC10703170; doi:10.3389/fmicb.2023.1282135)
Supplement: Supplementary file 2 [file Table_2.docx]

**Supplementary Table 2_ST2.** Primers and probes used for real-time multiplex PCRs for the identification of *Vibrio cholerae* non-O1/non-O139 strains.

GenBank numbers of reference sequences for *sodB and ctxA* genes are as followed: *Vibrio cholerae* NCTC8457 (GenBank AAWD01000215), and *Vibrio cholerae* strain B (GenBank AY376267).

GenBank numbers of reference sequences for *rfb* gene are as followed: *Vibrio cholerae* O:1- N16961 (GenBank LT906614.1), and *Vibrio cholerae* O:139- F9993 (GenBank CP046840.1).

| Target/gene | Primer/Probe | Sequence (5'–3') | Localization |
| --- | --- | --- | --- |
| *V. cholerae*  sodB | Vc-sodB-for  Vc-sodB-rev  Vc-sodB-TM | aagacctcaactggcggta  cagcaaaagaaccgaatgct  Cy5- gcaggtttggaaccacactt -BHQ-2 | 276-294  420-401  311-330 |
| *V. cholerae*  ctxA | Vc-ctxA-for  Vc-ctxA-rev  Vc-ctxA-TM | agttcattttggggtgcttg  ggaaacctgccaatccataa  FAM-catcgtaataggggctacagaga-BHQ-1 | 369-388  484-465  400-488 |
| *V. cholerae*  O:1 | Vc-O1-for  Vc-O1-rev  Vc-O1-TM | CCCAGATTGTAAAGCAGGATGG  TGAATGCGGTAGTGGGCTTA  Cy5-ACCTGGCCCACACACTTCTAGGTTCG-BHQ-2 | 258221-258196  258131-258152  258263-258244 |
| *V. cholerae*  O:139 | Vc-O139-for  Vc-O139-rev  Vc-O139-TM | AAGCGCTTAAAGCTCATCCC  AACACCCGGCTCTAACTTGA  FAM-ACGCCTGACGATTAATGCTGCCTTCA-BHQ-1 | 1590877-1590852  1590827-1590846  1590957-1590938 |
