# Supplementary material for: Virulence and resistance patterns of Vibrio cholerae non-O1/non-O139 acquired in Germany and other European countries
Source: Front Microbiol. 2023 Nov 22;14:1282135. doi: 10.3389/fmicb.2023.1282135 (PMC10703170; doi:10.3389/fmicb.2023.1282135)
Supplement: Supplementary file 4 [file Table_4.docx]

**Supplementary Table 4_ST4**: *V. cholerae* genomes included into the query genome to define the cgMLST target scheme. For each *V. cholerae* genome, the strain ID and the NCBI Accession numbers are provided.

| *V. cholerae* strain ID | NCBI Accessions |
| --- | --- |
| 10432-62 | NZ_CP010812.1 |
| 2010EL-1786 | NC_016445.1, NC_016446.1 |
| C5 | NZ_CP013301.1, NZ_CP013302.1 |
| CRC1106 | NZ_CP013305.1, NZ_CP013306.1 |
| E1162 | NZ_CP013309.1, NZ_CP013310.1 |
| E9120 | NZ_CP013313.1, NZ_CP013314.1 |
| FDAARGOS_223 | NZ_CP020408.1, NZ_CP020407.1 |
| IEC224 | NC_016944.1, NC_016945.1 |
| M2140 | NZ_CP013315.1, NZ_CP013316.1 |
| MJ-1236 | NC_012668.1, NC_012667.1 |
| NCTC5395 | NZ_CP013317.1, NZ_CP013318.1 |
| O395 | NC_012582.1, NC_012583.1 |
| I-1300 | NZ_CM003192.1, NZ_CM003193.1 |
